# THE ROLE OF METABOLIC SURGERY FOR PATIENTS WITH OBESITY GRADE I AND TYPE 2 DIABETES NOT CONTROLLED CLINICALLY

**DOI:** 10.1590/0102-6720201600S10025

**Published:** 2016

**Authors:** Josemberg CAMPOS, Almino RAMOS, Thomaz SZEGO, Bruno ZILBERSTEIN, Heládio FEITOSA, Ricardo COHEN

**Affiliations:** Interassociation guideline carried out by Brazilian Society for Bariatric and Metabolic Surgery (SBCBM), Brazilian College of Surgeons (CBC) and Brazilian College of Digestive Surgery (CBCD), São Paulo, SP, Brazil

**Keywords:** Metabolic surgery, Obesity, Type 2 Diabetes Mellitus, Bariatric surgery

## Abstract

**Introduction:**

Even considering the advance of the medical treatment in the last 20 years with new and more effective drugs, the outcomes are still disappointing as the control of obesity and type 2 Diabetes Mellitus (T2DM) with a large number of patients under the medical treatment still not reaching the desired outcomes.

**Objective:**

To present a Metabolic Risk Score to better guide the surgical indication for T2DM patients with body mass index (BMI) where surgery for obesity is still controversial.

**Method:**

Research was conducted in Pubmed, Medline, Pubmed Central, Scielo and Lilacs between 2003-2015 correlating headings: metabolic surgery, obesity and type 2 diabetes mellitus. In addition, representatives of the societies involved, as an expert panel, issued opinions.

**Results:**

Forty-five related articles were analyzed by evidence-based medicine criteria. Grouped opinions sought to answer the following questions: Why metabolic and not bariatric surgery?; Mechanisms involved in glycemic control; BMI as a single criterion for surgical indication for uncontrolled T2DM; Results of metabolic surgery studies in BMI<35 kg/m^2^; Safety of metabolic surgery in patients with BMI<35 kg/m^2^; Long-term effects of surgery in patients with baseline BMI<35 kg/m^2^ and Proposal for a Metabolic Risk Score.

**Conclusion:**

Metabolic surgery has well-defined mechanisms of action both in experimental and human studies. Gastrointestinal interventions in T2DM patients with IMC≤35 kg/m^2^ has similar safety and efficacy when compared to groups with greater BMIs, leading to the improvement of diabetes in a superior manner than clinical treatment and lifestyle changes, in part through weight loss independent mechanisms . There is no correlation between baseline BMI and weight loss in the long term with the success rate after any surgical treatment. Gastrointestinal surgery treatment may be an option for patients with T2DM without adequate clinical control, with a BMI between 30 and 35, after thorough evaluation following the parameters detailed in Metabolic Risk Score defined by the surgical societies. Roux-en-Y gastric bypass (RYGB), because of its well known safety and efficacy and longer follow-up studies, is the main surgical technique indicated for patients eligible for surgery through the Metabolic Risk Score. The vertical sleeve gastrectomy may be considered if there is an absolute contraindication for the RYGB. T2DM patients should be evaluated by the multiprofessional team that will assess surgical eligibility, preoperative work up, follow up and long term monitoring for micro and macrovascular complications.

## RESUMO

**Introdução:**

Mesmo considerando o avanço do tratamento clínico ocorrido nos últimos 20 anos, com novos e mais eficientes medicamentos, os dados ainda são desanimadores quanto ao controle da obesidade e da diabete melito tipo 2 (DMT2),com grande parcela de doentes em tratamento clínico ficando fora da meta desejada de controle.

**Objetivo:**

Apresentar proposta de Escore de Risco Metabólico para melhor orientar a indicação cirúrgica do diabete em pacientes com índice de massa corpórea (IMC) mais baixo nos quais o uso de procedimento cirúrgico para obesidade ainda é controverso.

**Método:**

Foi realizada pesquisa nas bases de dados Pubmed, Medline, Pubmed Central, Scielo e Lilacs entre 2003-2015 correlacionando os descritores:cirurgia metabólica, obesidade e diabete melito tipo 2. Adicionalmente, representantes das sociedades envolvidas emitiram opiniões em pontos nos quais não existia na literatura trabalhos com graus de evidência elevados.

**Resultados:**

Foram encontrados 45 artigos relacionados que foram analisados pelos critérios da medicina baseada em evidências*.* As opiniões agrupadas procuraram responder as seguintes questões: Porque cirurgia metabólica e não bariátrica?; Mecanismos envolvidos no controle glicêmico; IMC como critério isolado de indicação cirúrgica para o DMT2 não controlado; Resultados de estudos de cirurgia metabólica em IMC<35 kg/m^2^; Segurança da cirurgia metabólica em pacientes com IMC<35 kg/m^2^; Efeitos em longo prazo da cirurgia em pacientes com IMC inicial <35 kg/m^2^; Proposta de Escore de Risco Metabólico.

**Conclusão:**

A cirurgia metabólica tem mecanismos de ação bem definidos tanto em estudos experimentais quanto em seres humanos. As intervenções gastrointestinais em diabéticos com IMC≤35 kg/m^2^ possuem segurança e eficácia semelhantes aos grupos com IMCs maiores, levando a melhora do diabete de forma superior aos tratamentos clínicos e mudanças de estilo de vida, em parte através de mecanismos independentes da perda ponderal. Não há correlação entre o IMC inicial e perda ponderal em longo prazo com os índices de sucesso do tratamento cirúrgico. O tratamento cirúrgico é opção para os pacientes portadores de DMT2 sem adequado controle clínico, com IMC entre 30 e 35, após minuciosa avaliação seguindo os parâmetros dispostos no Escore de Risco Metabólico aqui proposto. DGYR é a técnica indicada para os pacientes selecionados no Escore, existindo a possibilidade de indicação da gastrectomia vertical para os casos em que exista contraindicação para ela. O paciente deve ser avaliado por equipe multiprofissional envolvida na indicação, preparo e acompanhamento após as operações e acompanhados com monitorização de complicações micro e macrovasculares.

## INTRODUCTION

The type 2 diabetes mellitus (T2DM) is characterized by defects in the insulin secretion and sensitivity. The resistance to its action is the initial phenomenon of the disease, gradually declining beta-cell function to emerge hyperglycemia^2^. Data released in 2015 by the International Diabetes Federation estimated that there are about 415 million diabetics worldwide, with growth potential to 642 million by the year 2040. Diabetes has been associated with 5 million deaths in 2015, surpassing the deaths from HIV, tuberculosis and malaria together for the same year. Brazil occupies the fourth position worldwide in number of diabetics, with 14.3 million recorded in 2015^21^.

Many clinical studies have shown the importance in DMT2 control to prevent complications of the disease and, so, improving life quality and reducing mortality^25^ ^,^ ^27^. However, the goals of this control in clinical practice are generally not achieved; only 27% of type 2 diabetics reach the goal of treatment, that is to have glycosylated hemoglobin (HbA1c) below 7%^5^. 

In a recent publication in USA, considering the three main objectives of clinical control (HbA1c<7%, LDL cholesterol<100 mg/dl and blood pressure less than 130x80 mmHg), only 18.8% were able to achieve the recommended values^43^.

Even considering the advancement of medical treatment occurred in the last 20 years with new and more effective drugs, the data is still discouraging with large portion of patients outside the desired control target. In addition, changes in lifestyle with adequate food standard, regular physical activity and weight loss - essential to achieve the disease control targets - are difficult to maintain in the long run^14^. In this scenario, metabolic surgery comes as effective to achieve lasting control of metabolic risk factors and promote proper weight loss, contributing to improved results in obesity grade I and T2DM^1^. It is noteworthy that for patients with T2DM and obesity grade II and III without proper control with medication, all medical societies involved in the treatment of diabetes and obesity have recognized bariatric surgery as the best alternative to disease control.

## METHODS

Survey was conducted in Pubmed, Medline, Pubmed Central, Scielo and Lilacs between 2003 and 2015 correlating the headings: metabolic surgery, obesity and diabetes mellitus type 2. In addition, representatives of the involved associations have issued opinions on points on which there were no high degree evidences in the literature.

## RESULTS

Were found 45 related articles that were analyzed by the criteria of evidence-based medicine

### Why metabolic surgery instead bariatric surgery? 

Bariatric surgery is indicated for the treatment of obese patients with body mass index (BMI) greater than 40 kg/m^2^ or for those with a BMI above 35 kg/m^2^, which comorbidities of difficult control, as T2DM, hypertension, dyslipidemia, sleep apnea, osteoarthritis and herniated disc, among others. Metabolic surgery can be defined as performing any surgical procedure involving the anatomical modification of the gastrointestinal tract resulting in better metabolic control of comorbidities induced by weight excess, such as T2DM.

A large number of publications with significant case series and randomized controlled trials with follow-up for several years showed good results in diabetes remission, even in patients with a BMI less than 35 kg/m^2^ ^1^. The T2DM remission rate varies according to the surgical procedure, being the best results observed in operations in which are associated gastric reduction and intestinal bypass, when compared with the purely restrictive techniques^35^. Thus, a large number of these procedures are being done each year in the world and gradually they are incorporated as part of the diabetes treatment algorithms, alongside changes in lifestyle and pharmacotherapy. Using the name "metabolic surgery" is not just semantics; however, it has the main objective to make clear that the surgical indication is not related to BMI only, but mainly in the normalization of metabolic disorders with improved quality of life. The main goals of metabolic surgery, in addition to weight loss, are metabolic control, with consequent cardiovascular risk reduction.

### Mechanisms involved in glycemic control

Several studies have shown that insulin sensitivity in morbidly obese patients, diabetic or not, improves with weight loss after operation^3^ ^,^ ^28^. Initially, the most widely accepted hypothesis to explain the metabolic effects were only related to weight loss. However over time, it has been shown that could happen improvement of glycemic control in the immediate postoperative period, even before significant weight loss, which showed clearly the involvement of other mechanisms beyond slimming^7^ ^,^ ^34^ ^,^ ^39^. Furthermore, when comparing a purely restrictive surgical technique (adjustable gastric band) with gastrojejunal derivation in Roux-en-Y (DGYR) even after similar weight loss, the percentage of diabetes remission is significantly higher with the second technique (17%vs.72%, p<0.001)^3^ ^,^ ^22^. When a comparison is made between obese patients that had the same weight loss after DGYR or low calorie diet, it is observed that control of diabetes is greater in the surgical group, with less need for antidiabetic medications and lower levels of postprandial glucose^3^ ^,^ ^22^.

Studies have shown that more than just calorie restriction and weight loss, intestinal rearrangement in some surgical techniques, as DGYR and biliopancreatic diversion are involved in rapid improvement of diabetes. Two hypotheses have emerged to explain these results: the "distal intestine" and the "proximal intestine". The first suggests that the arrival of nutrients less digested more quickly to the distal intestine stimulate the production of hormones that lead to glycemic control^36^. Mediators more accepted in this case are the incretin hormones, with action to stimulate insulin secretion and reduce the food intake^17^ ^,^ ^24^. In the second hypothesis, the duodenal exclusion in itself and also of the proximal jejunum from food transit prevents the secretion of a supposed sign that promote insulin resistance and T2DM. Recently, a study in mice demonstrated that jejunal proteins hinder the signaling of insulin in muscles, worsening insulin resistance^37^.

Ghrelin is a hormone produced in the stomach and duodenum and stimulates the secretion of other counter-regulatory insulin hormones, which is changed by DGYR. Despite the decreased production of this hormone seem be plausible explanation for the postoperative improvement of diabetes, studies are controversial and many of them did not show this reduction^15^ ^,^ ^44^.

Recent discussions also involve the intestinal microbiota as a regulator of metabolic mechanisms and imunoinflamatory axis connecting physiologically intestine, liver, muscles and brain^22^. Studies in rats and humans have shown differences in gut microbiota of obese and non-obese, and also in obese between pre and postoperative period of DGYR. The studies suggest that changes in intestinal microbiota play a role in the pathophysiology of obesity and metabolic bariatric surgery results^19^ ^,^ ^45^. However, more studies are needed to elucidate the matter.

### BMI as a single criterion for surgical indication for uncontrolled T2DM 

The indications for bariatric surgery in Brazil are based on the Resolution of the Federal Council of Medicine 2,131/15, recently published, which expanded the list of comorbidities accepted for indication^13^. Through it, it is indicated bariatric surgery for people with a BMI above 40 kg/m^2^ or above 35 kg/m^2^ with comorbidities related to obesity. However, these standards do not specifically emphasize the treatment of T2DM.

BMI is not a good tool for choosing the best treatment for diabetic patient since it does not reflect the distribution of adipose tissue and does not discriminate the differences in relation to race, gender, age and body composition^32^. Due to non-dependent antidiabetic mechanisms in weight loss or base BMI, surgery in diabetic grade 2 is being considered by several endocrinology and metabolic surgery associations, mainly because more than 50% of diabetic patients have BMI below 35 kg/m^2^ ^5^ ^,^ ^6^ ^,^ ^9^. Persistence of high rates of morbidity and mortality in diabetic patients is a sign that the answer to current treatments have not been effective. Faced with this reality, the metabolic surgery option should be considered in selected cases. The Swedish Obesity Subjects study showed that metabolic surgery has a preventive effect on the incidence of T2DM, particularly in patients with impaired fasting glucose, and initial BMI did not influence this preventive effect^10^.

Isolated anthropometric data do not seem to be the best factor for metabolic surgery indication in diabetic patients, as the best candidate would be the individual with increased insulin resistance, increased visceral and liver fat and high cardiovascular risk associated with BMI. For this reason, there is worldwide movement between experts in the field to change the surgical indication guidelines.

In 2011, the International Federation of Diabetes first introduced metabolic surgery in the T2DM. Also, treatment algorithms were introduced as alternative for patients with BMI between 30 and 35 kg/m^2^ with diabetes uncontrolled despite optimal drug-dealing, especially in the presence of other major risk factors for cardiovascular disease^16^. In 2014, the regulatory department of medical practice in the UK - National Institute for Health and Care Excellence - published its guidelines for the treatment of T2DM, and metabolic surgery was considered as part of the algorithm treatment fot those not compensated and with BMI>30 kg/m^2^ ^29^.

### Results of metabolic studies in surgery in BMI<35 kg/m **^2^**

Müller-Stich et al.^26^ published systematic review from studies directly comparing surgical vs clinical interventions in T2DM covering 818 participants. Each study concluded that several surgical procedures had superior outcome when compared to a variety of non-surgical interventions on the diabetes remission. The overall relative risk of the superiority of operations was 14.1 among all studies, and 22 among those who examined only patients with preoperative BMI<35 kg/m^2^. The overall mean HbA1c (%) fell by 1.5 points in postoperative compared with clinical treatment, and the first group of patients used less medications for diabetes in comparison to the clinic group.

Meta-analysis also recently published by Rao et al.^33^ examined the effects of DGYR on T2DM in nine publications, with a total of 343 subjects (BMI of 19-35 kg/m^2^, 1-7 years of follow-up). There was no mortality and rates of surgical complications were 6-20%, similar to the number reported in patients with IMC≥35 kg/m^2^. All nine publications have reported significant reductions in HbA1c after surgery with 2.8 points average reduction. In general, the procedure decreased fasting glucose levels of approximately 60 mg/dl more than the number of non-surgical interventions. The diabetes remission rate ranged from 65-93%, which is at least as high as the historically reported in patients with IMC≥35 kg/m^2^.

In systematic review of diabetes remission predictors after metabolic surgery it was seen that the overall remission rate was equivalent between 60 studies mean preoperative IMC≥35 kg/m^2^ as compared with 34 studies with mean preoperative BMI<35 kg/m^2^ (71% vs 72% respectively). The diabetes remission rates were also similar in each operation among patients with BMI higher vs lower than 35 kg/m^2^ (89% complete remission in biliopancreatic diversion, 77% in DGYR, 62% in adjustable gastric banding, and 60% for vertical gastrectomy). Surprisingly, among many basic characteristics of the patients, the only significant predictor of the magnitude of postoperative decrease in HbA1c was lower pre-operative abdominal circumference^31^.

In September 2015 it was held the Diabetes Surgery Summit II, which brought together several experts to define new guidelines for the indication of metabolic surgery independently of base BMI, but from 30 kg/m^2^. Thus, meta-analysis was performed on the event with 11 randomized clinical trials published directly comparing surgical vs non-surgical approaches for treatment of diabetes, including patients with BMI<35 kg/m^2^. All 11 studies reported superior results with surgery treatments for diabetes remission when compared to clinical treatments and/or glycemic control, with surgical superiority in ten of them. This is a unanimous evidence Level 1A, showing that surgery leads to improvement more significantly than diabetes drugs and lifestyle changes^18^.

As with diabetes remission outcomes and glycemic control, postoperative HbA1c levels reduction in comparison to clinical interventions is similar between studies in which groups had higher initial BMI or less than 35 kg/m^2^. This conclusion is clearly visible in STAMPEDE data, arguably the best randomized trial published to date. At all times over three years, surgical patients exhibited greater reduction in HbA1c compared to medical patients. This finding was similar among participants where the average baseline BMI was below or above 35 kg/m^2^ ^28^ ^,^ ^40^.

### Metabolic surgery safety in patients with BMI<35 kg/m **^2^**

The security in this group was examined in large systematic review published by the Agency for Healthcare Research and Quality in USA. Surgery caused greater BMI, HbA1c, blood pressure, LDL and triglycerides reductions than clinical interventions. Surgical adverse events were relatively low; surgical mortality was from 0.0 to 0.3%, similar to historical data for patients with BMI≥35 kg/m^2^; and most surgical complications were not serious, without requiring major interventions. Also it was seen that there was no excessive weight loss in these patients when used regulated techniques^12^.

### Long-term effects of surgery in patients with initial BMI of <35 kg/m **^2^**

The efficacy and safety of DGYR were studied prospectively in 66 patients with T2DM and BMI 30-35 kg/m^2^, followed over six years. This cohort had a mean duration of diabetes of 13 years, mean HbA1c of 9.7%, with 40% of insulin. However, there was a rapid decrease in mean HbA1c in the first months for non-diabetic levels, with subsequent maintenance of this degree of improvement in glycemia over six years. At the end of the study, 88% of participants with diabetes remained in remission (defined as HbA1c<6.5% without medication), clearly reached 11% improvement in diabetes, and only one remained unchanged. There was no relationship at any point of follow-up (from one month to six years) between the magnitude of weight loss and the degree of improvement of glycemic control. The blood pressure decreased during the study, and the total cholesterol, LDL cholesterol and triglycerides, while HDL cholesterol increased progressively for six years. These changes have led to significant improvement in eventual cardiovascular risk^11^.

A recent study by Hsu et al.^20^ showed similar results among patients in the Asia-Pacific region with T2DM and BMI<35 kg/m^2^. With follow-up greater than five years, they examined the antidiabetic effects of both DGYR and vertical gastrectomy compared to the clinical treatment in 351 patients. Despite efforts to get similar populations, the surgical group had HbA1c higher baseline average (9.1% vs. 8.1%) and longer duration of diabetes, introducing conservative biases against surgical superiority on the glycemia. However, HbA1c and BMI were both reduced to a much greater extent in the surgical group, and these changes were largely stable since the six months to five years.

The maintenance of HbA1c<6.5% free of drugs for diabetes was achieved in 64% of surgical patients compared with 3% of those with conservative treatment. At five years, the surgical group also exhibited greater reduction in abdominal circumference, central adiposity, LDL cholesterol, triglycerides and blood pressure. The mortality rate was statistically equivalent (1.9% after surgery, 3.0% after clinical treatment)^41^. These findings between the BMI of patients are comparable to long-term studies of metabolic surgery for individuals with T2DM and IMC≥35 kg/m^2^ ^4^ ^,^ ^8^ ^,^ ^23^ ^,^ ^41^.

### Proposal

**FIGURE 1:**
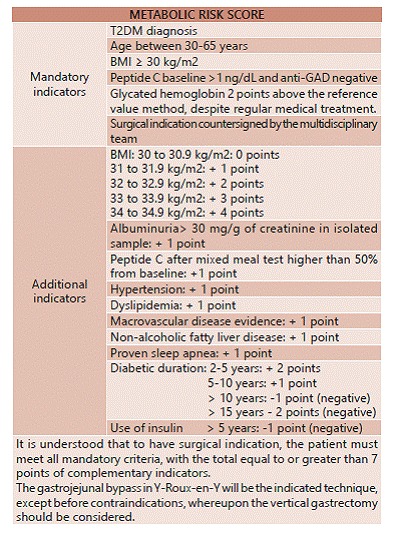
Metabolic risk score established by interassociation guideline carried out by the Brazilian Society of Metabolic and Bariatric Surgery (SBCBM), Brazilian College of Surgeons (CBC) and Brazilian College of Digestive Surgery (CBCD)

Based on scientific evidence showing similar results with those obtained in obese grades II and III, with low morbidity and mortality, and with evidence level 1 and 1A in short and long term, associations here represented propose the surgical treatment indications for T2DM not controlled using the Metabolic Risk Score (Figure 1).

## CONCLUSIONS

1) T2DM is chronic and progressive illness and in some situations is difficult to control with the best medical treatment and behavioral changes.

2) Metabolic surgery has well-defined mechanisms of action both in experimental and in humans studies. Gastrointestinal interventions in diabetics with IMC≤35 kg/m^2^ has similar safety and efficacy to groups with higher BMIs, leading to improvement in diabetes better than clinical treatments and lifestyle changes, in part through mechanisms independent of weight loss. There is no correlation between baseline BMI and weight loss in the long term with the success rate of surgical treatment.

3) Surgical treatment is option for patients with T2DM without adequate clinical control, with BMI between 30 and 35, after thorough evaluation following the prepared parameters in Metabolic Risk Score herein.

4) DGYR technique is indicated for patients selected by metabolic risk score, with the possibility of indicating the vertical gastrectomy for cases where there is a contraindication to its application.

5) The patient should be evaluated by multidisciplinary team of surgeons, clinicians, nutritionists and mental health professionals, if necessary, all of them participating in the indication, preparation, postoperative follow-up with monitoring of micro and macrovascular complications.
